# People Adapt to Personal Singular They Through Exposure

**DOI:** 10.1162/OPMI.a.366

**Published:** 2026-07-15

**Authors:** Yining Ye, Jennifer E. Arnold

**Affiliations:** Department of Psychology and Neuroscience, University of North Carolina at Chapel Hill, Chapel Hill, NC, USA

**Keywords:** pronoun comprehension, discourse processing, personal they, language adaptation, referential processing

## Abstract

Although *they* is often considered a plural pronoun (e.g., “Liz and Will went to the library. They borrowed a textbook”), it is increasingly used as a singular pronoun for individuals whose personal pronouns are *they*/*them* (e.g., “Alex went to the store. They bought a sandwich”), a usage we call *personal they*. This usage wasn’t broadly available until relatively recently, but its increasing frequency requires comprehenders to identify a singular referent when appropriate. What mechanism accounts for this change? While one possibility is that explicit awareness can boost consideration of the singular meaning of *they* (Arnold et al., 2021), here we test the hypothesis that comprehension facility can be facilitated through exposure. In two experiments, participants listened to stories where *they* referred to either a singular character (Alex) or to two characters. Participants were more likely to choose the singular interpretations after singular exposure, consistent with evidence that adaptation affected on-line processing in a mouse-tracking task (Ye & Arnold, 2025). Results support the role of exposure as a mechanism for the increased availability of singular *they* interpretations.

## INTRODUCTION

The prescriptive rule of English defines *they* as a plural pronoun that co-refers with multiple or a group of people (e.g., “The beach was full of visitors. They were waiting for the sunset.”). However, the use of *they* has long extended beyond this traditional form. For centuries, *they* has been used as a singular, generic pronoun to refer to nonspecific or gender-unknown antecedents (e.g., “Someone left their umbrella. They might come back for it”) in epicene contexts (e.g., Balhorn, 2004; Noll et al., 2018) or contexts where the referent’s identity is underspecified (Arnold, 2026b). We call this older usage *underspecified singular they*. More recently, *they* has also become widely used as a personal pronoun (e.g., “Alex went to the store. They bought a sandwich”). Often this choice reflects a nonbinary gender identity, but not always (Sanders, 2021). Because this usage is driven by the conscious adoption of *they* as a personal pronoun, we call it *personal they*.

Corpus and survey data indicate that the distribution and acceptability of singular *they*, both underspecified and personal *they*, are rapidly expanding in English (Balhorn, 2004; Camilliere et al., 2021; Conrod, 2019; Gustafsson Sendén et al., 2021; Konnelly et al., 2023), which reflects a broader social shift toward gender-inclusive language. What explains this change? We ask this question from a cognitive perspective. The use of *they* as a singular pronoun is more than just explicit claims to support it (for example, through social media posts). It also requires speakers to successfully implement its usage in both production and comprehension.

Changing uses of singular *they* have been explained as a change “from above” (Conrod, 2018), meaning a top-down change driven by social awareness and institutional support for inclusivity. This view suggests that one cognitive mechanism supporting processing fluency may be conscious choice and social support of the new form.

However, recent evidence suggests that change may also be supported through exposure, suggesting that cognitive mechanisms of priming and adaptation may also support community-level changes. Exposure to underspecified singular *they* increases the likelihood that people interpret ambiguous pronouns as singular (Kesan & Arnold, 2025), demonstrating that people may undergo an implicit adaptation to the changing use of *they* through experience. This raises a key question: is the rise of personal *they* primarily the result of intentional, socially motivated reform, or does it also reflect a more automatic adaptation to shifting patterns in linguistic input?

Understanding the cognitive basis of the changing use of *they* is essential, since personal *they* often challenges comprehenders’ existing grammatical expectations. Previous studies have found that singular *they* elicits a processing cost that is not observed for other common and frequent pronouns like plural *they*. For example, Leventhal et al. (2020) conducted an ERP study to test whether singular *they* is processed differently from other singular pronouns (e.g., *he*, *she*). Participants who were familiar with nonbinary gender identities read sentences with pronouns that either matched or mismatched the antecedent. If the pronoun mismatched the antecedent by gender (*Lillian … he*) or by using *they* in the singular sense (*Lillian … they*), ERP recordings showed a P600 compared to the matching cases (*Lillian … she* or *Lillian and Paul … they*). Thus, even for people who are familiar with nonbinary gender identities, they experienced processing difficulty when comprehending singular *they*.

Nevertheless, the use of personal *they* is rapidly increasing. Large-scale demographic and survey data show that more people now explicitly identify with and use *they*/*them* pronouns than ever before. For instance, a national survey by Minkin and Brown (2021) reported that about one in four U.S. adults personally know someone who uses *they*/*them* pronouns, which is a 8% increase since 2018. This parallels the rise in people who identify themselves as nonbinary, particularly among the younger generations (Herman et al., 2022; Wilson & Meyer, 2021). So what drives the increasing frequency of personal *they* in the language? One argument is that there is a strong social trend to avoid misgendering and promote respect for individuals’ pronoun choices. This increasing awareness makes personal *they* socially salient (Conrod, 2020).

In support of a role for a conscious adoption mechanism, a recent study by Arnold et al. (2021) showed that comprehension of personal *they* could be facilitated if there is explicit introduction about the pronoun use. In the study, participants were introduced to three characters (Alex, Liz, and Will) who use *they*, *he*, *she* pronouns respectively. Participants were assigned to conditions in which they were given either an explicit or implicit introduction. The explicit condition emphasized the pronoun each character used, while the implicit condition only gave the character names. Nevertheless, in both conditions participants were able to learn the pronouns of each character through training examples, and the authors ensured ceiling performance on learning that Alex uses *they*/*them* pronouns. Participants then read a two-sentence story, for example “Alex went running with Liz. They fell down,” and answered a question, e.g., “Who fell down?” The pictures were either one-character picture or two-character picture, representing a singular and a plural interpretation of the pronoun respectively. Results showed that participants assigned *they* to Alex more often when the pronouns had been introduced explicitly.

However, while explicit social cues can encourage people to use *personal they*, the intentional interpretation is unlikely to fully explain how a relatively new form becomes integrated into one’s language system, which operates rapidly through automatic and unconscious processes. To truly comprehend and produce personal *they* as part of fluent language use, individuals likely need substantial exposure and practice in supportive contexts that reinforce its meaning. Therefore, an additional possibility is that people learn to interpret and use personal *they* through exposure. Because pronoun comprehension is fairly automatic, it is dominated by the activation of the most plausible referent in the current context (e.g., Ariel, 1994; Arnold et al., 2000; Grosz et al., 1995; Kehler & Rohde, 2013; Stevenson et al., 1994). Given the much higher baseline frequency of plural they, a plural antecedent will usually win when it is available in the language context. Yet, with repeated and unambiguous exposure to personal *they* in contexts where it clearly refers to a definite person, the singular interpretation may become more accessible and begin to compete with the plural default. This leads to the hypothesis that the expanding use of personal *they* arises from people adapting to this pronoun form through exposure, a process known as referential adaptation.

### Referential Adaptation

Recent studies on referential adaptation provide a technique to test the hypothesis that people can adapt to personal *they* through exposure (e.g., Contemori, 2021; Contemori et al., 2021; Johnson & Arnold, 2023; Kaiser, 2009; Roy et al., 2025; Ye & Arnold, 2023). Referential adaptation occurs when frequent exposure to a referential pattern guides future pronoun comprehension by modulating existing discourse biases. For example, adults tend to follow a bias to assign pronouns to the subject of the prior sentence, such that they think *she* refers to *Ana* in *Ana saw Liz. She waved.* However, this bias can be changed with recent exposure. Johnson and Arnold (2023) used a referential adaptation paradigm where they first exposed participants to a series of unambiguous referential structures with pronouns that always referred to the subject (“Ana painted the wall with Will. She …”) or to the nonsubject (“Ana went to the library with Will. He …”). Participants were then tested on ambiguous pronouns, which assessed participants’ interpretation preferences (e.g., “Ana was cleaning the apartment with Liz. She …”). Questions assessed participants’ interpretation of the pronoun, and showed that people were more likely to assign the pronoun to the previous subject in the subject-exposure condition than the nonsubject-exposure condition. This referential adaptation paradigm successfully captures comprehenders’ tendency to follow the exposure pattern of referential structures, a robust finding that has been replicated several times (e.g., Arnold, 2026a; Kesan & Arnold, 2025; Roy et al., 2025; Ye & Arnold, 2023). While this adaptation paradigm uses repeated exposure to multiple items with the same referential pattern, other studies have found priming effects from exposure to a single prime (Contemori, 2021; Contemori et al., 2021; Kaiser, 2009).

Overall, research on referential adaptation provides compelling evidence that recent exposure affects pronoun comprehension. This suggests that even through adulthood, people are sensitive to the frequency of referential patterns in local contexts and dynamically shift their processing biases based on recent experience.

This paradigm offers an approach for testing whether ongoing changes to the use of *they* in English are influenced by the frequency of exposure to singular vs. plural uses. If it is, frequently hearing *they* used in the singular may encourage people to consider the singular interpretation in ambiguous contexts. We can apply this paradigm by exposing comprehenders to contexts where *they* clearly refers to one person. Following exposure, we can test their pronoun comprehension in ambiguous contexts where *they* can be either singular or plural, to see if their interpretation preference shifts. If exposure guides interpretation, more singular examples should yield more singular interpretations.

Support for this prediction comes from a recent study on the older use of singular *they*, which we call “underspecified *they*”. Kesan and Arnold (2025) asked participants to read repeated stories that either always used *they* in the singular (*the cyclist … they*) or the plural (*the cyclists … they*). They asked whether this exposure would guide the rate of assigning *they* to a singular referent in test stories like “The gardener showed the flowers to the man. Then they went to wash up.” Results showed that indeed, people assigned a singular interpretation more often in the singular exposure condition. However, notably this type of singular *they* is different from the use of personal *they*, because it is an older and more familiar form. The question in the current study is whether similar priming affects the interpretation of personal *they*.

Early evidence on this question suggests that it might be difficult to observe priming for personal *they*. Arnold et al. (2021) tested how explicit pronoun introduction, discourse cues and exposure influence comprehenders’ interpretations of *they*. Both explicit introduction and supportive discourse cues, such as placing the character using personal *they* in the first-mentioned position, made singular interpretations more likely. However, even when participants received double the number of training stories using personal *they*, they did not become more likely to interpret *they* as singular over the course of the study. This pattern suggests that comprehension of personal *they* depends on explicit pronoun introduction and supportive discourse cues, but may be relatively resistant to momentary priming. Also, Arnold et al. (2024) examined whether repeated exposure to personal *they* during a storytelling task would make speakers more fluent or more likely to produce it. They manipulated both discourse cues, such as whether the target character appeared alone or with another character, and exposure (reading personal *they* in the story prompt). Although discourse cues reliably shaped pronoun use, exposure did not make speakers more likely to produce personal *they*, nor did it reduce the rate of misgendering errors. However, both of these manipulations were relatively weak. Thus, previous work does not answer our question about whether priming affects personal *they* interpretation.

In the current study we use a stronger priming manipulation than previous studies to test whether priming affects the interpretation of personal *they*. We examine the interpretation of singular *they* in contexts that include Alex, who uses *they*/*them* pronouns. This makes personal *they* contextually available in addition to the plural interpretation. This mimics real-world contexts where one person is known to use *they*/*them* pronouns.

Answering this question is critical because it provides a real-life window onto understanding the relation between priming and long-term language learning. One major theory of adaptation is implicit learning (Bock & Griffin, 2000; Chang et al., 2006; Fine & Jaeger, 2013). On this theory, exposure to unexpected structures leads to long-term changes in the comprehension system such that these structures are relatively more available. While this theoretical approach was developed for syntactic priming, it clearly predicts that there is a link between short-term exposure and long-term language learning. If we can observe short-term priming in an experimental session, it opens the possibility that priming plays a role in ongoing changes in English-speaking communities where personal *they* is used.

In the context of the current study, we use exposure to refer to the repeated linguistic input participants receive during the experiment, and *priming* or *adaptation* to refer to the resulting shift in later interpretations. Although these concepts are related, they are not identical. Prior work on referential adaptation shows that repeated exposure to a referential pattern can influence later pronoun interpretation (e.g., Johnson & Arnold, 2023, Ye & Arnold, 2023), while other studies show that even a single recent prime can yield priming effects (e.g., Contemori, 2021; Contemori et al., 2021; Kaiser, 2009). One broader theoretical possibility is that such effects reflect implicit learning, where exposure gradually changes the comprehension system and increases the availability of particular structures or interpretations (e.g., Bock & Griffin, 2000; Chang et al., 2006; Fine & Jaeger, 2013). However, the current study does not determine how much exposure is needed to shift interpretation, nor whether the adaptation effect reflects the most recent prime, the cumulative distribution of local input, or both.

### Interpretation Preference vs. Processing Fluency

If exposure affects the interpretation of singular *they*, we would expect it to both change the final interpretation and also change the online processing that occurs as comprehenders try to resolve it. Preliminary evidence suggests that indeed it does.

Previous studies have found a processing cost elicited by singular *they* pronouns referring to a definite referent (e.g., Leventhal et al., 2020). This processing cost remains even with the explicit pronoun introduction. Arnold et al. (2023) conducted a mouse-tracking study to examine the differences in online processing of singular versus plural *they* in an object-identification task. The study also explicitly introduced the pronouns of the story characters (Alex (they), Liz (she), and Will (he). Participants read three-sentence stories shown as the examples below and were presented with three pictures. The critical stories always had Alex and one of the other two characters Liz or Will. Alex was manipulated to be either first- or second-mentioned in the story. The corresponding pictures were then Alex, the other character or both together. Under each picture were two objects, only one was mentioned in the spoken story. Participants were asked to click on the mentioned object, which was manipulated to be placed under the picture of either Alex alone or both characters together. These object locations under different character pictures indicated a singular and a plural interpretation of the *they* pronoun. During the process, participants’ reaction times and mouse-trajectories were recorded for further analysis.

(1)  ***Alex as first-mentioned***: Alex and Liz were cleaning up after a dinner party. Alex snatched a towel from Liz. Then they dried the plates.
(2)  ***Alex as second-mentioned***: Liz and Alex were cleaning up after a dinner party. Liz handed a towel to Alex. Then they dried the plates.

The study compared the differences in online measures between the singular and plural interpretation conditions as well as the secondary effect of Alex’s order-of-mention. Reaction times were longer in the condition where the target object was placed under Alex, which means that people were slower in clicking the object that indicated a singular interpretation. One of the mouse-tracking measures examined x-flips (i.e., the directional changes of the mouse along the horizontal axis on the screen) that occurred following the pronoun, which measured the degree of competition among available choice candidates during processing. X-flips were more frequent in the singular condition, in which the target object was placed under Alex. This suggested that people seemed to experience a stronger competition when the target location was inconsistent with their expectations based on a plural interpretation of *they*. However, these effects were modulated by order-of-mention, such that the difference between conditions was strongest when Alex was the second-mentioned, possibly because people had to overcome both the dominant plural interpretation of *they* and the first-mention bias. Overall, this study provided important evidence for online processing of singular *they*, that people with explicit pronoun introduction still experience difficulty processing this low-frequency pronoun.

Based on the above findings, it’s clear that singular *they* is more difficult to process than other singular pronouns like *he* and *she*, and also than plural *they*. Although the explicit introduction alone increases the salience of the pronoun status, it is not enough to eliminate the processing cost of singular *they* to make it compete with the dominant plural *they*.

This raises a question about whether exposure could also modulate the differences in processing the different usages of *they*. If it does, adaptation to either singular or plural *they* would inhibit the processing of the other pronoun use. In principle, strong adaptation might even overturn the dominance status of plural *they* within the experiment, such that participants might experience difficulty processing plural *they* after repeated exposure to singular *they*.

Supporting this possibility, a mouse-tracking study (Ye & Arnold, 2025) provided initial evidence for competition between singular and plural *they* following exposure. This study used the same mouse-tracking paradigm as Arnold et al. (2023). Participants were exposed to unambiguous stories containing either singular or plural *they*, disambiguated by visual cues, and were then tested on unambiguous critical stories. Mouse-tracking data revealed that participants experienced greater competition when processing plural-*they* stories following singular-*they* exposure, and the reverse pattern following plural-*they* exposure. This was reflected in greater mouse deviation and more horizontal-axis flips, meaning their cursor paths strayed further from a direct route and changed left-right direction more often, both indicators of increased uncertainty between options. However, there were no effects of adaptation on either reaction time or area-under-the-curve (AUC), which is another measure of overall cursor curvature. Although the effects were not fully consistent across measures, these findings offer preliminary evidence that exposure can modulate real-time processing of *they*, influencing how comprehenders resolve competition between its singular and plural interpretations. Using a similar paradigm, the current study tests ambiguous context in order to examine both which interpretation is selected (singular or plural) and the speed with which it is made.

### The Current Study

**Figure 1.**
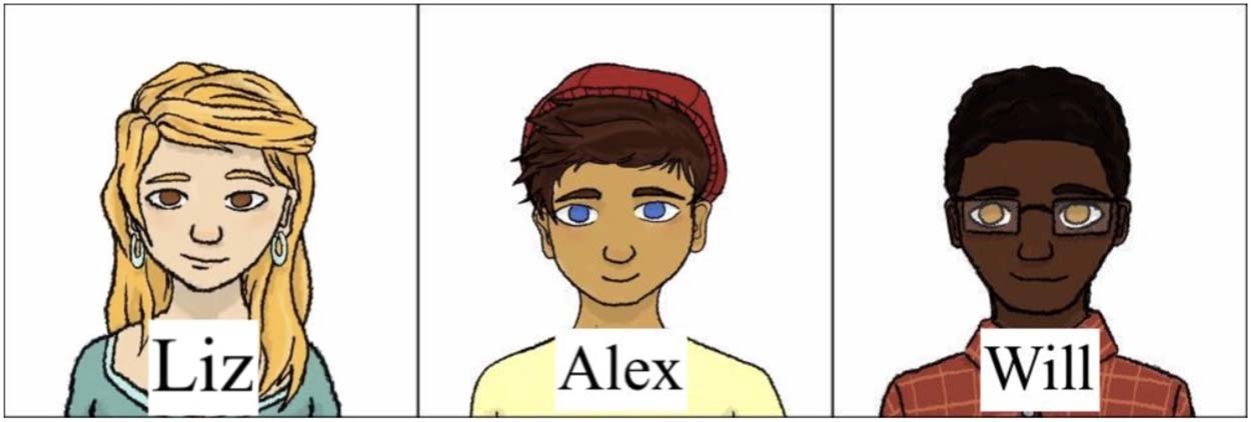
Character pictures presented with instructions “Meet these people—Alex, Liz, and Will. Alex uses they/them pronouns, Liz uses she/her pronouns, and Will uses he/him pronouns.”

The primary objective of the current study was to examine whether the understanding of personal *they* can be boosted by exposure. That is, in stories like “Will gave some water to Alex. Then they put down the batons,” do people assign *they* to Alex (vs. Will-and-Alex) more often if they have recently heard multiple examples of singular they? Across two experiments, we examined personal *they* adaptation in young adults who are current undergraduate students and likely to have experience with this pronoun form. For both studies, participants were explicitly introduced to the pronouns used by the characters, shown in Figure 1 below. We integrated the referential adaptation paradigm from prior studies (Johnson & Arnold, 2023; Roy et al., 2025; Ye & Arnold, 2023) to manipulate exposure conditions within the object-clicking paradigm from Arnold et al. (2023) and Ye and Arnold (2025).

**Figure 2.**
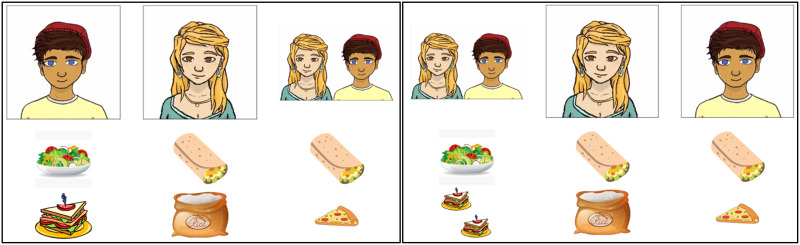
Sample displays for the exposure stories. Singular exposure (left) has the target object (sandwich) under Alex (“Liz and Alex ordered lunch at the cafeteria. Liz showed the menu to Alex. Then they asked for a sandwich”). Plural exposure (right) has the target (sandwiches) under plural picture (“Liz and Alex ordered lunch at the cafeteria. Liz showed the menu to Alex. Then they asked for some sandwiches”).

Both experiments examined whether exposure shifts comprehenders’ behavioral tendency to interpret *they* as either singular or plural. We used the same task as Ye and Arnold (2025), except that for the critical items, the visual display was consistent with two interpretations— both singular and plural. For each trial, participants listened to the story and were presented with three character pictures (for example, Alex, Liz, and Alex-and-Liz) and a pair of objects underneath each character picture (stimuli example shown as Figure 2 below). Participants were told that the objects were the ones each character interacts with, and they should click on the target object they hear mentioned in the story.

**Figure 3.**
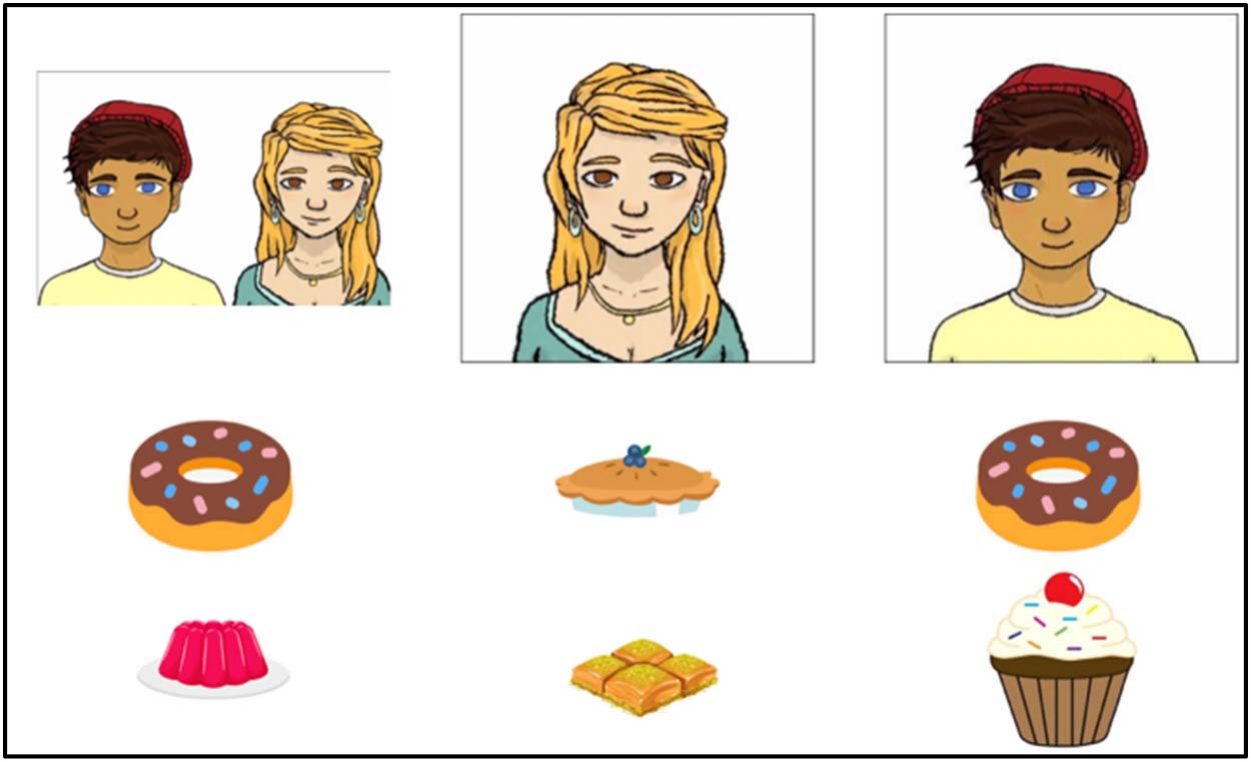
Sample display for an Alex-only exposure story, in which only Alex is mentioned in the spoken story (“Alex wanted to get a treat. The ice cream store was closed. Then they decided to get a cupcake instead”). As in all trials, the display contains three character pictures, including one plural picture. The target object appears under Alex. Although alternative objects also appear under Liz and the plural character picture, those interpretations are not supported because Liz is not mentioned in the story. Across Alex-only items, Liz and Will appeared equally often in the non-target individual picture. Alex appeared equally often in the left and right positions.

Participants were exposed to 24 unambiguous exposure stories in which the pronoun *they* always referred to either Alex or two characters (e.g., Alex-and-Liz) as a between-subject manipulation. Therefore, in the exposure stories the pronoun was disambiguated by the target object location. For the singular exposure condition, all exposure stories had a target object placed under the picture of Alex, implying a singular interpretation. For the plural exposure condition, all exposure stories had a target placed under the picture of two characters together, implying a plural interpretation. The number of character pictures was held constant across all trials. The visual displays all had three character pictures (two pictures with one person each and one with the two together), but the stories varied in discourse contexts. Sample displays for the exposure stories were shown in Figures 2 and 3. Details are discussed in the Materials and Design section.

**Figure 4.**
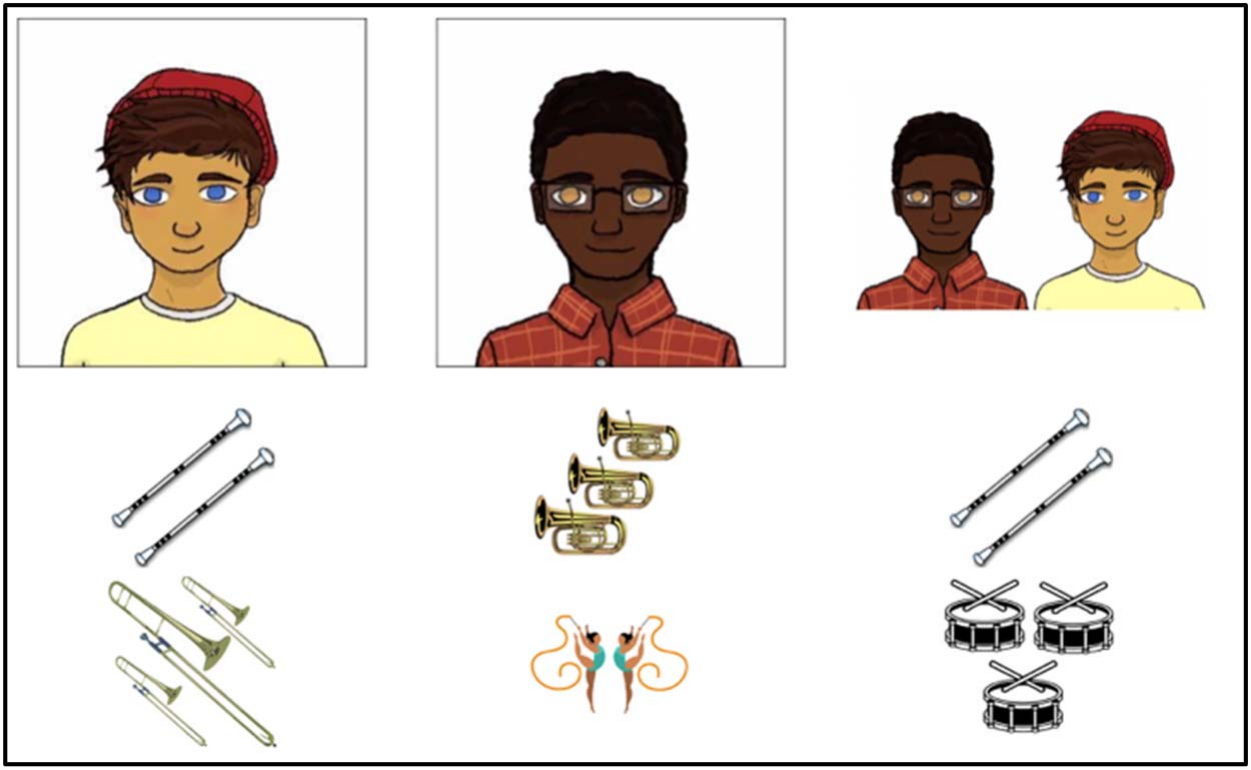
Sample display for a critical ambiguous story (“Will and Alex were at marching band practice. Will gave some water to Alex. Then they put down the batons”). Two identical targets are placed under both Alex and the plural picture to measure pronoun comprehension.

The critical stories, in contrast, were ambiguous because two identical object pictures were placed under both Alex and the plural picture (stimuli example shown as Figure 4 below). This required participants to draw their own interpretation. Choosing the object under Alex indicated that participants adopted a singular interpretation for the pronoun while choosing the object under the plural picture indicated a plural interpretation. Picture selections were used to measure pronoun comprehension tendencies following exposure. In addition, 20 filler stories were intermixed with the exposure and critical stories using the same stimuli structure, but they don’t have Alex involved in any stories.

In the exposure stories, the visual display supported a single interpretation of *they* (either singular or plural) for all items in a list. Repeatedly selecting one interpretation was expected to increase its accessibility on later critical trials. We hypothesized that participants would be more likely to pick targets under Alex in critical stories following singular exposure than following plural exposure.

Experiment 1 was conducted in a laboratory on undergraduate students from the University of North Carolina at Chapel Hill. Experiment 2 tested Prolific workers who were also college students to replicate the finding with a broader population of undergraduate students.

## EXPERIMENT 1

### Methods

#### Participants.

82 undergraduate students from the University of North Carolina at Chapel Hill participant pool participated in exchange for course credit. Posted participation criteria were that participants should be native speakers of English and at least 18 years old. One participant was excluded from further analysis for not meeting the minimum age requirement. We examined target selection to ensure that all participants selected the correct target on at least 75% of the unambiguous stories; no participants were excluded for this reason. 81 participants were included in the final analysis with an average age of 18 (range: 18–22). 40 participants received the singular exposure condition, and 41 participants received the plural exposure condition.

#### Materials and Design.

We used linguistic stimuli adapted from Arnold et al. (2023). A total of 64 stories included 24 exposure items, 20 critical items and 20 fillers. Each story consisted of two context sentences introducing two characters, followed by another sentence with a pronoun. We used two types of exposure stories in each condition in order to create a set of exposure items that all shared either the singular or plural interpretation but varied in structure (see Table 1). For the singular exposure stories, 12 were dubbed “Alex-only” items, where Alex was the only person mentioned, once by name and twice with “they”. 12 were “Alex-second” items, where Alex was mentioned second in a sentence with Will or Liz (e.g., *Will dug a hole with Alex. Then they planted a tree*). The pronoun “they” was disambiguated because the display only had one target item under Alex. Thus, the Alex-only exposure items strongly favored the singular interpretation, while the Alex-second items imposed an ambiguity that was resolved in the singular. Critically, this design meant that the singular pronoun “they” was resolved to the prior subject half the time and to the prior prepositional object half the time. Thus, the exposure effect was not confounded with an overall first-mention bias. This prevents the possibility that any priming effects could reflect subject-antecedent exposure priming, as has been observed elsewhere (e.g., Johnson & Arnold, 2023).

**Table 1.** Sample stimuli from main experiment.

**Item Type**	**Exposure Type**	**Stimuli**
Exposure	Singular (Alex-only)	Alex went to the store. They looked through all the aisles. Then they bought some milk.
	Singular (Alex-second-mentioned)	Will and Alex were gardening. Will dug a hole with Alex. Then they planted a tree [picture resolves to singular *they*].
	Plural (Liz-Will)	Will and Liz went to the store. They looked through all the aisles. Then they bought some milk.
	Plural (Alex-second-mentioned)	Will and Alex were gardening. Will dug a hole with Alex. Then they planted a tree [picture resolves to plural *they*].
Critical	/	Liz and Alex were at a restaurant. Liz handed the menu to Alex. Then they ordered some wine.
Filler	/	Will and Liz went to a farm. Will saw the cows with Liz. Then she fed the goats.

For the plural exposure stories, 12 included a plural antecedent “Liz and Will” or “Will and Liz”, which was unambiguously plural. The other 12 used the same Alex-second structure as in the singular exposure condition, but in this case the display disambiguated the pronoun to the plural interpretation. This variation of story contexts was used to emphasize the uniform interpretations across different contexts and thus maximize the support for adaptation.

The critical stories had Alex with either Liz or Will and the fillers only had either Liz, Will or both. The critical stories were ambiguous, such that both singular and plural interpretations were possible based on the story and on the picture, since the critical object appeared below both Alex and the plural picture. This set-up was used for probing participants’ own interpretations of “they”. By contrast, exposure stories had unambiguous interpretations of *they*. The Alex-only and Liz-Will exposure stories were unambiguous based on the text. The Alex-second items were ambiguous in the text, but the intended resolution of the ambiguity was manipulated through the location of target objects in visual displays. Sample stimuli are shown below in Table 1. The full list of stimuli is reported in Appendix A.

For all stories, the visual display presented three pictures of characters placed at top-left, top-middle, and top-right respectively. Two of these were pictures of individual characters and one showed two characters together. Two object pictures were placed under each of the character pictures respectively. The visual objects were thematically related to the sentence but only one target object was mentioned in the spoken story. In the exposure items, the target object was placed under either Alex or the plural picture to indicate the interpretation of *they*. In the critical stories, there were two duplicates of the critical picture, one under Alex and one under the plural picture. For consistency, we also had duplicate pictures for the exposure and filler stories, but these were never mentioned.

For exposure stories, which were unambiguous, there is only 1 target object. For example, in a singular prime, the target mentioned at the end is unique, placed under Alex only. Therefore, the exposure items provided one interpretation of *they*, either singular or plural. We expected this manipulation to promote either the singular or plural interpretation through repeated exposure. In these stories, the identical objects are distractors, placed under the other two character pictures.

For critical stories, which were ambiguous, the identical objects were placed under Alex and the plural-character picture, allowing the target to be associated with either Alex or the plural characters. This required participants to choose which character they think is more likely the referent of “they”. Participants’ target selections indicated their interpretation preferences.

The main task began with 4 consecutive exposure stories to establish the representation of the exposure interpretation. The other items were intermixed to avoid a cluster of consecutive items of the same types. For all stories, the picture locations were counterbalanced, such that Alex and the plural pictures were placed on left or right the same amount of time. The fillers had target character pictures appearing left, middle or right at an approximately equal frequency.

### Procedure

The experiment was implemented on the internet via the PC-Ibex platform, beginning with a background questionnaire, which asked about age, sex, gender identity, ethnicity/race, language experience, language proficiency and report of language disorders. Following the background questionnaire, participants were introduced to three characters and were explicitly told about their pronoun use (Figure 1). An example visual display was presented to familiarize participants with the overall setup. Participants were told that they would be listening to short stories while viewing the character pictures. Under each character picture there were two objects, which are the ones the characters interact with. They were required to click on the object they hear mentioned in the story. Before the main task, two practice trials with accuracy feedback were given. Participants then proceeded to the main task where no accuracy feedback was provided. Participants’ reaction times for each trial and target selections were recorded for further analysis.

### Analytical Approach

Our pre-registered analysis examined the rate of selecting the singular pronoun interpretation as a function of our exposure predictor. As an exploratory analysis, we also examined response times as a function of exposure and response type (singular vs. plural).

We first examined whether any trials should be excluded for (1) responses made substantially before the offset of the critical word, indicating inattentiveness or an attempt to rush through the task, and/or (2) incorrect selections (i.e., choosing a distractor). RTs were calculated relative to the offset of the critical word, so negative values indicate responses made before the critical word had finished. After reviewing the negative RT values, we excluded trials with responses made 1,000 ms earlier than the critical word offset. This cutoff was chosen post hoc to exclude only responses that occurred well before the end of the critical word and were therefore unlikely to reflect processing of the critical disambiguating information, while retaining responses made shortly before the critical word offset. Under this criterion, one trial was excluded for a response made 3,924 ms earlier than the critical word offset, since this response likely occurred before the participant had fully heard the critical information needed to resolve the pronoun. Another trial with negative RTs close to the critical word offset (−1 ms) was retained. No responses were excluded for incorrect selection of distractors.

### Results: Pronoun Comprehension

We analyzed the results using a mixed-effects logistic regression in SAS proc glimmix with a binomial distribution and a logit link. The primary predictor variable is Exposure condition (Singular coded as .5 vs. Plural coded as −.5). The dependent variable was whether the target selections in the critical stories indicated a Singular Interpretation (coded as 1) or a Plural Interpretation (coded as 0). A basic model that included Exposure as the only predictor was conducted to test its main effect on pronoun interpretation outcomes. Random intercepts were included for subjects and items. Attempts to fit a maximal random-effects structure, including random slopes, resulted in non-convergent or singular models. Therefore, the final model retained random intercepts only.

As an exploratory analysis, we also conducted two additional analyses to examine potential effects of target location and order. To assess whether response patterns varied as a function of where the target pictures appeared, one model included Exposure, Horizontal Target Location (targets on the left coded as .5 vs. targets on the right coded as −.5) and Vertical Target Location (targets on top coded as .5 vs. targets at the bottom coded as −.5), and all corresponding interaction terms as predictors. To test whether the likelihood of giving a singular vs. plural response changed over the course of the experiment, a second model included Exposure, Order and their interactions as predictors. Both the exploratory models included random intercepts for subject and item only because models with a maximal random-effects structure did not converge.

#### Exposure Effect.

**Figure 5.**
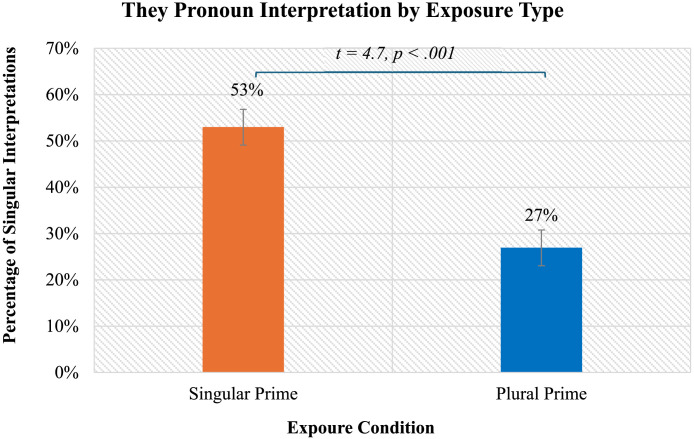
Experiment 1: Singular interpretation rates by exposure condition. Bars represent standard error of participant means.

Results revealed a main effect of exposure on pronoun interpretations. Summarized in Figure 5, the average rate of singular interpretations is 27% among participants given plural exposure and 53% among participants given singular exposure. Singular interpretations were significantly higher in the singular exposure condition (*t* = 4.7, *p* < . 0001). Model estimates were reported below in Table 2.

**Table 2.** Experiment 1: Inferential statistics for percentage of singular responses by exposure.

**Effect**	**Estimate (St. Error)**	***t* Value**	***p***
Intercept	−0.68 (0.23)	−2.96	<.01
Singular_Exposure	1.65 (0.35)	4.7	<.0001

#### Exposure and Target Location Effects.

In addition to the main effect of exposure, a significant effect was found for target locations, such that participants showed a general right-side bias for target selections. Across exposure conditions, the rate of singular interpretations was higher when targets were placed on the right side of the screen (*t* = −2.11, *p* < .05), but the Vertical Target Position (top vs. bottom) did not have a significant effect on response patterns. Model estimates are summarized in Table C1 in Appendix C.

#### Exposure and Order Effects.

In addition to the main effect of exposure, there was a marginal interaction of Exposure and Order (*t* = 1.82, *p* = .069). This pattern suggests a weak tendency for the exposure effect to increase slightly over the course of the experiment, consistent with possible adaptation or learning over time. However, this trend was only marginally significant.

### Results: Reaction Times

For RT analyses, in addition to the previous exclusions for comprehension responses, we further excluded trials where participants took an unusually long time to respond. 65 responses were excluded for reaction times longer than 2 standard deviations above average (*M* = 2623.27).

For all included data, we analyzed the results using a mixed-effects logistic regression in SAS PROC MIXED. The primary predictor variables were Exposure condition (Singular coded as .5 vs. Plural coded as −.5) and Response Type (Singular coded as .5 vs. Plural coded as −.5). The dependent variable was a continuous outcome of RTs in milliseconds. A basic model tested the effects of Exposure, Response Type and their interactions on RTs. A maximal random slope structure was used.

Again, as exploratory analyses, two additional models were used to assess whether RTs varied as a function of Target Locations, Order, and their interactions with Exposure and Response Type. All models used a maximal random slope structure.

#### Exposure and Response Type.

**Figure 6.**
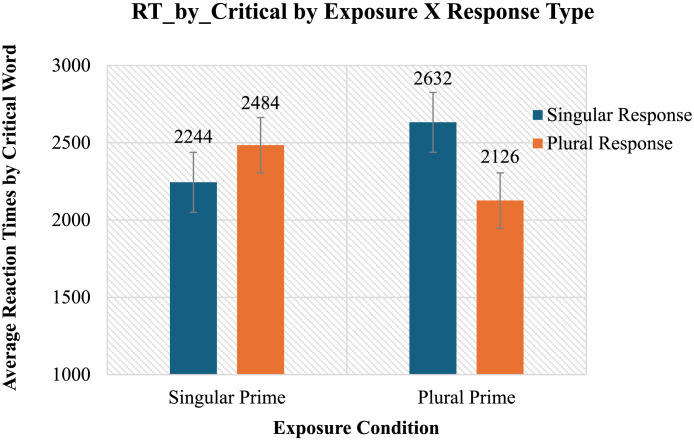
Experiment 1: Reaction times for singular interpretations as a function of exposure (singular vs. plural prime) and response types (singular vs. plural response). Bars represent standard error of participant means.

Reaction time analyses revealed a significant interaction between exposure condition and response type (see Table 3). Singular interpretations were made more quickly than plural interpretations following singular exposure while the pattern reversed in the plural exposure condition (Figure 6). This indicated that exposure differentially affected processing speed for singular vs. plural interpretations.

**Table 3.** Experiment 1: RT analysis results of linear mixed effects regression.

**Effect**	**Estimate (St. Error)**	***t* Value**	***p***
Intercept	2291.18 (165.90)	13.81	<.0001
Singular_Response	203.64 (201.40)	1.01	0.3146
Singular_Prime	201.67 (132.41)	1.52	0.1340
Response × Prime	−338.85 (160.03)	−2.12	0.0400

#### Exposure, Response Type and Target Location.

This model revealed no main effect of any of the predictors or their interactions. Target Location appeared unlikely to influence how long people took to respond. All model estimates are summarized in Appendix C, Table C3.

#### Exposure, Response Type and Order.

The model revealed a main effect of Response Type (*t* = 2.85, *p* < .05), with singular responses taking longer compared to plural responses across exposure conditions. This pattern suggests that even when a singular response was given, the decision might require greater processing effort, consistent with the lower baseline frequency of singular *they* compared to plural *they*. There was a significant main effect of Order (*t* = −3.65, *p* < .01), indicating that overall response times decreased across trials. In addition, a significant interaction of Order and Response Type (*t* = −2.34, *p* < .05) showed that RTs decreased more on singular responses than on plural responses over time. Notably, there was no interaction with Exposure, suggesting that the speeded responses over time was not different by exposure condition. All model estimates are summarized in Table C4.

### Discussion

Overall, we observed that participants were more likely to adopt the singular interpretations of *they* pronouns if they recently were exposed to this form of pronoun usage in the local context. The exposure, therefore, modulates interpretation of personal *they* beyond any facilitation from the explicit pronoun introduction. In addition, we observed a significant Exposure × Response interaction in RTs, suggesting that exposure may speed the selection of a matching response. This is the first demonstration that priming affects interpretation of personal *they*, and contrasts with previous failures to find such an effect (Arnold et al., 2021).

One open question is whether this effect is limited to the particular experimental conditions, namely that it occurred in-lab with an experimenter present, and in a university community where personal *they* is well known and socially supported. Participants were university students and the experimenters were also students and thus peers. Would the same effect occur in a remote setting without a co-present experimenter?

## EXPERIMENT 2

We tested this question with a new sample of participants from Prolific. We aimed to recruit a set of participants who, similar to those in Experiment 1, were at least somewhat familiar with personal *they*. We therefore limited the pool to young adults who self-identified as college/university students in the US or UK. Experiment 2 was not pre-registered, but it used the same sample size, analysis plan and predictions as for Experiment 1.

### Methods

#### Participants.

80 Prolific workers participated and were compensated $3.33 for a 20-min study session. Participants were required to be native speakers of English, reporting learning English before age 6 and with a native level of proficiency. The study was only accessible to participants who reported on Prolific that they (a) were living in the US/UK, (b) used English as their primary language, (c) had no literacy difficulties, hearing difficulties, or language disorders, (d) were a current undergraduate student and (e) were aged between 18 and 28. An additional 14 participants participated but did not complete the study, which automatically terminated at the 4th missed attention check (details described in the Procedure section below). All participants in the final sample met the criterion of at least 75% accuracy on target selection across unambiguous stories. The final sample included 80 participants with an average age of 22 (range: 18–28). 40 participants received the singular exposure condition, and 40 participants received the plural exposure condition.

#### Materials and Design.

The materials were identical to those in Experiment 1. The only modification in main task design was the target location. In Experiment 1, the targets appeared in the top-left and bottom-right corners twice as often in the top-right and bottom-left corners. To avoid potential bias from this imbalance, we counterbalanced target location in Experiment 2, such that the targets appeared equally often in all four possible positions (topL, topR, botL, botR).

### Procedure

The task itself was identical. The procedure was different in the following ways. First, the PC-Ibex script was distributed through Prolific, an online cloud-sourcing platform. Only participants who met our eligibility requirements had access to the study. Upon accepting the study, participants were redirected from Prolific to the PC-Ibex platform to proceed. Without an experimenter monitoring the study process, we implemented an attention check by calculating the number of errors in object selection on the first 75% of the unambiguous stories. The study would automatically terminate at the 4th incorrect object selection, and participants were asked to return the incomplete study on Prolific.

Additional pre- and post-survey questions were implemented to explore participants’ knowledge and experience with personal *they* (see Appendix B for details). Although this was not the focus of our analyses, exploratory analyses were conducted to see if experience correlated with responses and response times.

### Analytical Approach

We first examined whether any trials should be excluded for unusual response performance. For pronoun interpretation analyses, we excluded seven trials with incorrect selections (i.e., choosing a distractor), and six trials with responses occurring more than 1,000 ms before the critical word offset (range: −4,932 to −3,695 ms), using the same criterion in Experiment 1. Another five trials with negative RT values (range: −793 to −12 ms) were made briefly before the critical word offset and were retained. For the RT analyses, an additional 57 responses were further excluded for unusually long reaction times, defined as values exceeding 2 standard deviations above the mean (*M* = 2,513.25 ms).

### Results: Pronoun Comprehension

The models, variables and effects coding were identical to those in Experiment 1, except that in this analysis all models included a maximal random effects structure.

#### Exposure Effect.

**Figure 7.**
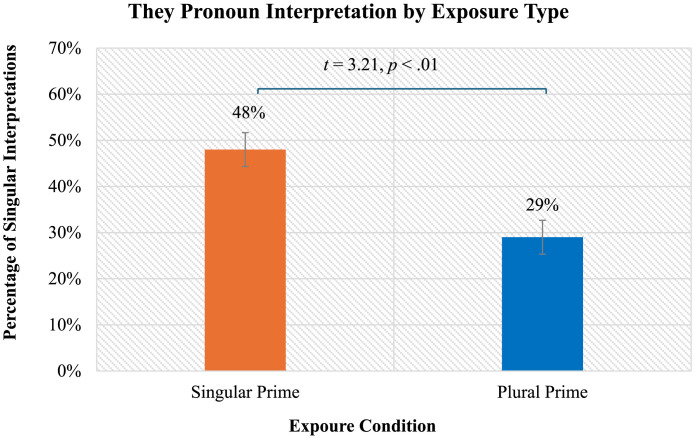
Experiment 2: Singular interpretation rates by exposure condition. Bars represent standard error of participant means.

Results revealed a main effect of exposure on pronoun interpretations. Summarized in Figure 7, the average rate of singular interpretations is 29% among participants given plural exposure and 48% among participants given singular exposure. Singular interpretations were significantly higher in the singular exposure condition (*t* = 3.21, *p* < . 01). Model estimates were reported in Table 4.

**Table 4.** Experiment 2: Inferential statistics for percentage of singular responses by exposure.

**Effect**	**Estimate (St. Error)**	***t* Value**	***p***
Intercept	−0.64 (0.19)	−3.29	<.01
Singular_Exposure	1.02 (0.32)	3.21	<.01

#### Exposure and Target Location.

This model revealed a main effect of Exposure (*t* = 3.32, *p* < .05). There were no significant main effects of Horizontal Target Location (*t* = − .48, *p* = .63) or Vertical Target Location (*t* = − .43, *p* = .67). However, there was a significant Exposure × Vertical Target Location interaction (*t* = −1.99, *p* < .05) and a marginal three-way interaction among Exposure, Horizontal, and Vertical Target Location (*t* = −1.89, *p* = .058). These interaction patterns were not consistent with our findings from Experiment 1, and did not align with our theoretical predictions. All model estimates were summarized in Appendix C, Table C5.

#### Exposure and Order.

The model revealed no significant main effects of Exposure (*t* = 1.07, *p* = .30) or Order (*t* = 1.24, *p* = .21). However, there was a marginal interaction between Exposure and Order (*t* = 1.96, *p* = .051), suggesting a weak tendency for the exposure effect to become stronger over the course of the experiment. This pattern may reflect gradual adaptation or learning, where repeated exposure to singular or plural *they* slightly modulated participants’ response tendencies as the task progressed. Model estimates are reported in Appendix C, Table C6.

### Results: Reaction Times

#### Exposure and Response Type.

**Figure 8.**
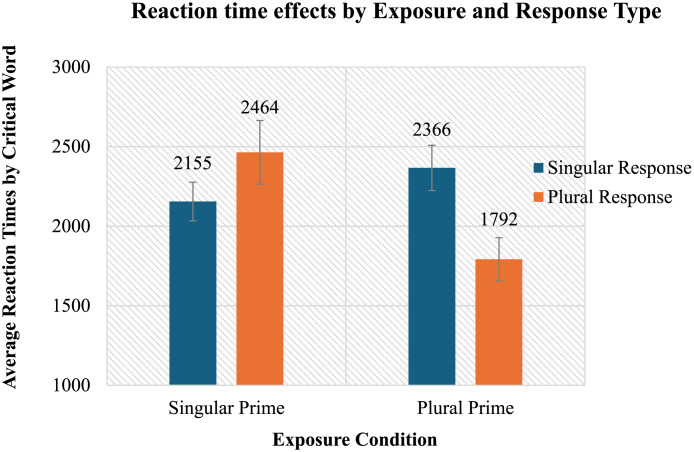
Experiment 2: Reaction times for singular interpretations by exposure and response types. Bars represent standard error of participant means.

Reaction time analyses revealed a significant interaction between Exposure Condition and Response Type (Table 5). Singular interpretations were made more quickly than plural interpretations following singular exposure while the pattern reversed in the plural exposure condition (Figure 8). This indicated that exposure differentially affected processing speed for singular vs. plural interpretations. This suggests that, in contexts without singular examples, comprehenders can default to a plural use of *they*. Even if they might be aware that *they* can be used as a singular pronoun for the character Alex, selecting the singular can incur additional time costs, potentially reflecting competition from the dominant plural *they*.

**Table 5.** Experiment 2: RT analysis results of linear mixed effects regression.

**Effect**	**Estimate (St. Error)**	***t* Value**	***p***
Intercept	1957.71 (166.16)	11.78	<.0001
Singular_Response	181.67 (146.44)	1.24	0.2202
Singular_Prime	587.29 (206.79)	2.84	0.0056
Response × Prime	−476.09 (171.09)	−2.78	0.0072

#### Other Models.

Additional exploratory analyses assessed the effects of Target Location and Order in addition to Exposure and Response Type on RTs. Similar to what we found in Exp. 1, no Target Location effects on RTs were observed. For the model including Order, we observed a marginal effect of Exposure Condition, a significant effect of Response Type, and a significant effect of Order. All model estimates were summarized in Tables C7–C8.

### Exploratory Analysis on Personal *They* Knowledge and Experience

As an exploratory analysis, we examined four^1^ self-report measures indexing participants’ knowledge and experience with personal *they*, including familiarity with nonbinary/gender-queer individuals, knowledge of *they*/*them* pronouns, self-reported use, and frequency of hearing *they*/*them* in everyday contexts. For one participant who selected “Do not wish to report” on familiarity, only that familiarity code was treated as missing. All other responses were retained. Each measure was standardized (*Z*-scored) and averaged to create a composite Familiarity predictor.

Two mixed-effects models were fit. The first examined the effect of Familiarity on singular/plural responses, and the second tested the effects of Familiarity and its interaction with Exposure and Response Type on reaction times. For responses, no reliable effect of Familiarity was observed. The RT model revealed a main effect of Exposure (*t* = 2.29, *p* < .05), as well as a nearly significant Exposure × Familiarity interaction (*t* = −1.99, *p* = .051).

To probe the nearly significant interaction, we conducted a simple-slope analysis treating Familiarity as a continuous moderator. Familiarity significantly predicted faster RTs in the Singular Exposure condition (*t* = −2.06, *p* < .05), but not in the Plural Exposure condition. This indicates the possibility that participants with greater familiarity and experience with personal *they* processed stimuli more efficiently overall when they had been exposed to the singular use of *they*, regardless of whether their eventual response was singular or plural. The result suggests that greater familiarity may facilitate adaptation to singular uses of they, leading to generally reduced processing effort in contexts where this usage is frequent. However, because the effect of Familiarity did not interact with Response Type, the familiarity effect may reflect general processing ease rather than faster singular interpretations. Future work should test whether familiarity helps people adapt to singular *they* or choose responses more quickly.

### Discussion

In sum, Experiment 2 replicated the robust exposure effect on pronoun comprehension from Experiment 1. Participants were more likely to adopt the singular interpretations if given singular exposure than plural exposure. For reaction times, we observed a competition effect among participants who received plural exposure. Singular selections were slower than plural selections in contexts without support for practicing singular use of personal *they*. This finding aligns with the initial evidence found in Ye and Arnold’s (2025) mouse-tracking experiment, in which participants experienced stronger competition for singular interpretations following plural exposure. However, in the mouse-tracking experiment, this RT asymmetry did not reach significance in the singular-exposure group. Taken together, the findings suggest that exposure can modulate real-time processing of *they*. Plural exposure reliably preserves a processing benefit for plural they and may even suppress the accessibility of singular interpretations. In contrast, the benefit of singular exposure appears less robust and may only modestly reduce the processing cost of singular they. Further research is needed to determine the extent to which exposure facilitates online comprehension of the singular form.

## GENERAL DISCUSSION

The current study examined referential adaptation to personal *they*. We asked whether people adapt to personal *they* through exposure, and explored whether adaptation influences processing fluency as well as final interpretation. In two experiments we found a clear and robust effect of exposure on pronoun interpretation: Participants exposed to personal singular *they* were more likely to adopt a singular interpretation for critical stories than those exposed to plural *they*.

Reaction times provided support for the role of exposure on singular *they* interpretation. In both experiments we observed an Exposure × Response interaction, indicating that exposure modulated processing speed as a function of what interpretations people adopted. We interpret this pattern to suggest that exposure to the singular usage makes the singular interpretation more available, and exposure to the plural usage makes the plural interpretation more available, and this availability facilitates the selection of the exposed form.

Our exploratory models suggested that the effects of exposure were not entirely static but showed subtle changes over the course of each experiment. In both experiments, we observed marginal Exposure × Order interactions, giving a hint that the influence of singular vs. plural *they* exposure on response selections may gradually strengthen as participants progress through the task. Although these tendencies were modest, they are consistent with the idea that exposure-based adaptation can accumulate over time, even within a relatively short experimental session.

The exploratory RT analyses further revealed that singular interpretations became faster over the course of the study, and RTs for singular responses decreased more steeply than those for plural responses. This pattern may reflect increasing familiarity or efficiency in selecting the less frequent singular interpretation once participants had encountered examples of it, in both exposure conditions. In the plural exposure condition, taking the singular interpretation in the critical items may have constituted a type of exposure in and of itself. Personal singular *they* is still such a low frequency form, even encountering it a few times may be enough to speed responses. Repeated exposure within the experiment may have allowed participants to adapt to singular *they*, gradually reducing its processing cost.

In Experiment 2, the exploratory analyses also provided marginal evidence that processing was shaped by participants’ broader familiarity and experience with personal *they*. Participants with greater prior experience tended to show more efficient processing, specifically in the singular-exposure condition. This pattern suggests that individual differences in knowledge and real-world personal *they* usage may facilitate adaptation to singular *they* during online comprehension.

But notably, exposure still modulated responses even for participants with prior familiarity with personal *they*. Indeed, the effect of familiarity had no effect on the rate of singular responses, nor did it interact with the prime manipulation. This lack of a finding may index the current state of personal *they* in English-speaking communities. Even in a young-adult student population, taking the singular interpretation of *they* is still relatively hard, even for people with relatively high familiarity. This finding is in line with other studies that have reported only modest effects of familiarity (e.g., Arnold et al., 2021).

Additionally, we found different effects of target location in each study, but overall this seems to suggest that the exposure manipulation worked by directing attention toward one interpretation over the other, and this may have heightened attention to stimuli that were more available because of their spatial position.

In sum, exposure to the singular, personal *they* increases the availability of the singular interpretation in competition with the plural interpretation of *they*. Thus, adaptation to local frequencies of singular versus plural *they* guides comprehenders’ assumptions about whether “they” is being used in a sense of singular or plural.

### Implications

#### Adaptation vs. Intentional Acceptance.

A key question concerns the mechanism driving the ongoing change in English pronoun usage. Sociolinguistic perspectives have often characterized the rise of personal *they* as a top-down shift motivated by social awareness, advocacy, and the institutional promotion of gender-inclusive language (e.g., Conrod, 2018). While social awareness is important for promoting this linguistic change by encouraging the acceptance and use of this linguistic form, it might not explain how the change is integrated in one’s language system. Even though participants in the current study were explicitly told that one of the story characters Alex uses *they*/*them* pronouns, which provides a strong top-down cue, robust differences in interpretation outcomes and reaction times emerged as a function of repeated exposure, above and beyond any effects of the explicit instruction. This suggests that adaptation operates at a level distinct from deliberate reasoning about social norms. If adaptation is guiding real-life changes in the acceptance of personal *they*, it would be consistent with implicit learning theories (e.g., Bock & Griffin, 2000; Chang et al., 2006), which suggest that exposure to linguistic forms can facilitate processing both in the short term and have lasting long-term effects.

Critically, the longer response times for singular interpretations in plural exposure provided an important hint about difficulty making an intentional decision to pick the singular interpretations. Even when participants intentionally allowed a singular interpretation, probably because they knew that Alex uses *they*/*them*, they still experienced processing competition between the dominant plural meaning and the singular meaning. This competition slowed them down, revealing that awareness alone does not guarantee automatic processing fluency. Instead, the language system seems to require sufficient experience-based calibration before singular *they* becomes as efficient to process as the plural form.

Perhaps surprisingly, prior familiarity with personal *they* had no effect on the rate of singular responses, and even though responses were faster for people with greater familiarity, they were equally fast for plural and singular responses. This suggests that interpretations are most strongly driven by the linguistic context, which in our stimuli promoted the plural interpretation. It is also possible that at this point in time, most people, including young adult students, do not have extensive experience with this form. Our familiarity data (Appendix D) show that 21% of our Exp. 2 participants report never hearing or reading personal *they*, and 36% encounter it only on occasion. Only 21% report hearing it a few times a week, and only 5% hear it every day. While we didn’t collect data for these questions in Exp. 1, another study in our lab did ask these questions from participants from the UNC psychology subject pool, and found a very similar rate of familiarity. Until and unless personal *they* is used with regularity, recent exposure may play an important role in facilitating the availability of singular interpretations.

#### Questions for Future Research.

The findings from these experiments establish that singular/plural exposure does affect the interpretation of they, and establish a paradigm for testing further questions about the mechanisms underlying this effect.

##### What Representations Are Tracked?

One question is about what representations people are keeping track of during the adaptation process. In our paradigm, when participants repeatedly encountered *they* referring clearly to a single person, this experience could have activated multiple linguistic representations. First, it is possible that participants were adapting to the lexical meanings of “they”, representing singular *they* vs. plural *they* as either separate lexical words with different meanings or separate senses of the same word. In this case, through singular exposure, the singular meaning of *they* becomes more accessible, increasing the likelihood of selecting that interpretation in ambiguous contexts. A second possibility is that people may have experienced structural adaptation, in which people activated the types of referents most likely to be referred to—that is, the singular-antecedent vs. plural-antecedent mappings. After repeated exposure to examples where *they* unambiguously refers to one person, people may have learned that *they* was more frequently linked to singular antecedents, and therefore made more singular interpretations. Adaptation to a certain type of antecedent may have been represented in terms of antecedent frequency or participants could have learned a disambiguation routine that is driven by the singular/plural distinction.

Exposure in the current paradigm might also have served as a reminder that *singular they* is acceptable and intended in the experimental context, leading to a reinforcement (or suppression) of singular *they* as a social norm. Relatedly, exposure to the singular may have reminded people that Alex uses they/them pronouns. Although the current findings cannot determine exactly which of these representations and processes is driving the adaptation, the exposure effect on both behavioral outcomes and reaction times suggests that the language system is flexible at resolving *they* as singular when the context supports it.

##### What Is the Time Course of Priming?

The current findings also leave open important questions about the mechanism and time course of the priming effect. Although we observed that repeated exposure shifted subsequent interpretation, we do not know how much input is necessary to produce this effect, or whether comprehenders are primarily influenced by the most recent prime or by the overall distribution of singular and plural uses across the discourse context. Addressing this question will help clarify whether adaptation to *personal they* is best characterized as an immediate priming effect, cumulative distributional learning, or an interaction between the two.

##### Does Frequency of Character Mention Impact Exposure?

Another question is whether exposure effects are impacted by the frequency of character mention. A natural consequence of our design was that Alex was mentioned far more in the singular-exposure than plural-exposure contexts. This type of confound is likely to occur in real life as well, especially for people who only know a few individuals with the personal pronouns *they*/*them*. In many situations, there is only one person who could be the referent of personal *they*. Is singular interpretation promoted by just talking about that person a lot, or specifically using *they* in the singular?

Our design provides one method of testing this question, because we used two types of exposure stories. In the “Alex-second” stories, the story was identical across exposure conditions but the pronoun was disambiguated by the visual display. In these items, Alex was mentioned in both exposure conditions, so character mention could not account for any exposure effect—as long as we assume that the priming effect is immediate and temporary. To test this, we restricted the analysis to critical trials immediately preceded by an Alex-second exposure item. We found that the exposure effect remained significant in both experiments (See Appendix C, Tables C12 and C13). This rules out the possibility that recent mention of Alex automatically activates the character’s representation and leads to the priming effect. However, if exposure effects are instead due to an assessment of the most frequent reference pattern (e.g, “singular *they* is common in this situation”), further work is needed to rule out a character-mention effect.

##### Are Participants Aware of the Exposure Pattern?

An additional uncertainty is how participants experienced the exposure trials, since no trial-by-trial feedback was provided during the main task. The exposure items provided a single interpretation of *they* across all items in a list, but the present design does not determine the extent to which participants consciously recognized that interpretation as the intended one. This may have varied across participants depending on their familiarity with personal *they*, their engagement with the task, or their sensitivity to the discourse context. Regardless, the findings do show evidence of priming, such that prior exposure shifted subsequent interpretation preferences.

### Conclusions

To our knowledge, our study provides the first experimental evidence that comprehenders adapt to personal *they* through recent, local exposure. This provides a mechanism for shifting pronoun interpretation. Recent input, even if not aligning with cumulative language experience, can reshape people’s expectations for language use. The current study provides evidence for the hypothesis that language changes as a function of experience, and provides a starting point for future research on how socially emerging forms of language develop and changes over time. It also motivates future research on quantifying how much exposure is needed, how long the shift lasts, and how broadly it transfers to new names and contexts.

## ACKNOWLEDGMENTS

Thank you to Michela Acosta, Jacob Bastian and Chance Mckinnie for assisting in running the experiment. Also, many thanks to Nicholas Michael Payst and Gabrielle Wells Garner for recording the instruction audios and spoken stimuli.

## FUNDING INFORMATION

This study was funded by NSF grants 1917840 and 2438559 to J. Arnold.

## DATA AVAILABILITY STATEMENT

Experiment 1 was pre-registered (https://doi.org/10.17605/OSF.IO/4K6AW). Data and code for both experiments are available at https://osf.io/wv24r.

## Note

^1^ The post-experiment questionnaire included five questions. We did not include the item “How many individuals with a non-binary gender identity do you know?” in our summary metric because it was very similar to the familiarity question, but less specific.

## APPENDIX A: EXPERIMENTAL STIMULI

**Table A1.** Stimuli for the singular exposure conditions.

**Item**	**Story**	**Story Type**
Practice	Alex and Liz went ice skating. Alex had to go back home. They forgot their skates.	Alex_only
Practice	Will and Liz took a waltzing lesson. They twirled around the room for an hour. Then they bumped into the table.	Will_Liz
Exposure	Alex went to the store. They looked through all the aisles. Then they bought some milk.	Alex_only
Exposure	Alex wanted to get a treat. The ice cream store was closed. Then they decided to get a cupcake instead.	Alex_only
Exposure	Liz and Alex ordered lunch at the cafeteria. Liz showed the menu to Alex. Then they asked for a sandwich.	Alex_second
Exposure	Will and Alex were having lunch at the park. Will passed a fancy box to Alex. Then they ate a piece of sushi.	Alex_second
Exposure	Alex was building some furniture. The instructions seemed impossible. Then they realized there was a missing screw.	Alex_only
Exposure	Alex decided to start a band. There were two spots left. They offered to play the xylophone.	Alex_only
Exposure	Alex decided to go for a hike. The trail had a warning about bears. Worried, they bought some bear spray.	Alex_only
Exposure	Alex moved into a new apartment. The air conditioning broke. They decided to buy a fan.	Alex_only
Exposure	Alex was in the mailroom. They picked up the mail. Then they borrowed scissors to open a package.	Alex_only
Exposure	Liz and Alex went to the zoo. Liz wanted to show the antelopes to Alex. But they got distracted by the penguins.	Alex_second
Exposure	Liz and Alex planned a fun outing at the lake. Liz went kayaking with Alex. They lost their oars.	Alex_second
Exposure	Will and Alex went to the amusement park. Will handed Alex a ticket. Then they bought cotton candy.	Alex_second
Exposure	Will and Alex were getting ready to go to the beach. Will packed beach supplies with Alex. Then they realized they forgot their sunscreen.	Alex_second
Exposure	Liz and Alex were getting ready to go shopping. Liz brought the coupons to Alex. Then they wrote a shopping list.	Alex_second
Exposure	Alex was playing video games. Suddenly the power shut off. Then they lit a candle.	Alex_only
Exposure	Alex was scrolling through social media. They realized their phone was on low battery. They plugged it into an outlet.	Alex_only
Exposure	Alex was baking a cake. They mixed all the ingredients in a bowl. Then they poured the batter into a pan.	Alex_only
Exposure	Alex was going to watch a movie. The popcorn was too expensive. Then they bought french fries instead.	Alex_only
Exposure	Alex was watching a TV show. Then the doorbell rang. They paused the show with the remote.	Alex_only
Exposure	Liz and Alex went to a concert. Liz was dancing with Alex. Then they took videos with their phone.	Alex_second
Exposure	Will and Alex were gardening. Will dug a hole with Alex. Then they planted a tree.	Alex_second
Exposure	Will and Alex decided to throw a party. Will drove Alex to the store. They bought lots of balloons.	Alex_second
Exposure	Will and Alex were bowling. Will loaned some shoes to Alex. Then they picked up two bowling balls.	Alex_second
Exposure	Liz and Alex were in an art class. Liz passed some pigments to Alex. Then they started painting on a canvas.	Alex_second
Critical	Will and Alex were at marching band practice. Will gave some water to Alex. Then they put down the batons.	
Critical	Will and Alex went grocery shopping. Will gave the grocery list to Alex. Then they grabbed a bag of chips.	
Critical	Liz and Alex went fishing. Liz handed a fishing pole to Alex. Then they got out the bait.	
Critical	Liz and Alex went to the farmer’s market. Liz gave a basket to Alex. Then they picked out some tomatoes.	
Critical	Liz and Alex sorted candy after Halloween. Liz passed a chocolate bar to Alex. Then they ate lollipops.	
Critical	Will and Alex went to play soccer. Will handed some cones to Alex. Then they put on their jerseys.	
Critical	Will and Alex went hiking. Will lent a backpack to Alex. Then they packed some snacks.	
Critical	Liz and Alex were studying together. Liz handed some notes to Alex. Then they looked through the textbook.	
Critical	Will and Alex went to a restaurant. Will handed the salt to Alex. Then they ate pasta.	
Critical	Liz and Alex were at a restaurant. Liz handed the menu to Alex. Then they ordered some wine.	
Critical	Will and Alex were deciding what food to make for a party. Will gave an apron to Alex. They made a cake.	
Critical	Liz and Alex went to the store. Liz gave the cart to Alex. Then they bought some soda.	
Critical	Will and Alex were pouring drinks. Will gave a cup to Alex. Then they had some orange juice.	
Critical	Liz and Alex went to a New Year’s Eve party. Liz gave a drink to Alex. Then they popped party poppers.	
Critical	Will and Alex were at the train station. Will gave some money to Alex. Then they bought two tickets.	
Critical	Liz and Alex joined a pottery club. Liz gave some clay to Alex. Then they made vases.	
Critical	Will and Alex were shopping for clothes. Will showed a cool jacket to Alex. Then they decided to buy hats instead.	
Critical	Liz and Alex went to a bakery. Liz lent some money to Alex. Then they bought croissants.	
Critical	Will and Alex were looking at the bookshelves. Will passed an encyclopedia to Alex. Then they rearranged the bookends.	
Critical	Liz and Alex were cleaning up after a dinner party. Liz handed a towel to Alex. Then they dried the plates.	
Filler	Will and Liz wanted to exercise. Will went with Liz to the gym. He ran on the treadmill.	
Filler	Liz and Will played chess. Liz was losing to Will. He captured the king piece.	
Filler	Liz and Will went to a corn maze. Liz rode a tractor with Will. She drank hot chocolate afterwards.	
Filler	Will and Liz went to a farm. Will saw the cows with Liz. Then she fed the goats.	
Filler	Will was fast asleep. Suddenly his alarm went off. He rushed to find his car keys.	
Filler	Liz went to a bar after work. The day had been exhausting. She ordered a cocktail.	
Filler	Will and Liz were at rehearsal. Will helped Liz set up her music stand. Then he got out the sheet music.	
Filler	Will and Liz were bored. Will played a prank on Liz. She sat on a balloon.	
Filler	Will was getting ready for a job interview. He practiced in front of a mirror. Then he put on a tie.	
Filler	Liz was driving home from work. It was foggy and hard to see. She swerved to avoid a deer.	
Filler	Will and Liz went to the airport. Will went through security with Liz. Then he got some coffee.	
Filler	Liz and Will went to the pool. Liz jumped in the water with Will. Then he put on his goggles.	
Filler	Liz and Will were in class. Liz discussed the lecture topics with Will. Then she sharpened a pencil.	
Filler	Will and Liz went to an art museum. Will admired some photography with Liz. She liked the paintings better.	
Filler	Liz was shopping at a jewelry store. None of the gift sets were affordable. So she only bought a necklace.	
Filler	Liz was taking a flight. The pilot warned about some turbulence. She put on some headphones to distract herself.	
Filler	Liz and Will joined an orchestra. Liz practiced with Will. He broke one of the strings on his cello.	
Filler	Liz and Will were doing some chores. Liz spent all day cleaning the apartment with Will. She loved using the new vacuum.	
Filler	Will was knitting a sweater. It was looking a bit too small. He grabbed some more yarn.	
Filler	Will was folding laundry. A spider crawled nearby. He hit it with a shoe.	

**Table A2.** Stimuli for the plural exposure conditions.

**Item**	**Story**	**Story Type**
Practice	Alex and Liz went ice skating. Alex had to go back home. They forgot their skates.	Alex_only
Practice	Will and Liz took a waltzing lesson. They twirled around the room for an hour. Then they bumped into the table.	Will_Liz
Exposure	Will and Liz went to the store. They looked through all the aisles. Then they bought some milk.	Will_Liz
Exposure	Liz and Will wanted to get a treat. The ice cream store was closed. Then they decided to get a cupcake instead.	Will_Liz
Exposure	Liz and Alex ordered lunch at the cafeteria. Liz showed the menu to Alex. Then they asked for a sandwich.	Alex_second
Exposure	Will and Alex were having lunch at the park. Will passed a fancy box to Alex. Then they ate a piece of sushi.	Alex_second
Exposure	Will and Liz were building some furniture. The instructions seemed impossible. Then they realized there was a missing screw.	Will_Liz
Exposure	Liz and Will decided to start a band. There were two spots left. They offered to play the xylophone.	Will_Liz
Exposure	Will and Liz decided to go for a hike. The trail had a warning about bears. Worried, they bought some bear spray.	Will_Liz
Exposure	Liz and Will moved into a new apartment. The air conditioning broke. They decided to buy a fan.	Will_Liz
Exposure	Will and Liz were in the mailroom. They picked up the mail. Then they borrowed scissors to open a package.	Will_Liz
Exposure	Liz and Alex went to the zoo. Liz wanted to show the antelopes to Alex. But they got distracted by the penguins.	Alex_second
Exposure	Liz and Alex planned a fun outing at the lake. Liz went kayaking with Alex. They lost their oars.	Alex_second
Exposure	Will and Alex went to the amusement park. Will handed Alex a ticket. Then they bought cotton candy.	Alex_second
Exposure	Will and Alex were getting ready to go to the beach. Will packed beach supplies with Alex. Then they realized they forgot their sunscreen.	Alex_second
Exposure	Liz and Alex were getting ready to go shopping. Liz brought the coupons to Alex. Then they wrote a shopping list.	Alex_second
Exposure	Liz and Will were playing video games. Suddenly the power shut off. Then they lit a candle.	Will_Liz
Exposure	Will and Liz were scrolling through social media. They realized their phones were on low battery. They plugged them into an outlet.	Will_Liz
Exposure	Liz and Will were baking a cake. They mixed all the ingredients in a bowl. Then they poured the batter into a pan.	Will_Liz
Exposure	Will and Liz were going to watch a movie. The popcorn was too expensive. Then they bought french fries instead.	Will_Liz
Exposure	Liz and Will were watching a TV show. Then the doorbell rang. They paused the show with the remote.	Will_Liz
Exposure	Liz and Alex went to a concert. Liz was dancing with Alex. Then they took videos with their phone.	Alex_second
Exposure	Will and Alex were gardening. Will dug a hole with Alex. Then they planted a tree.	Alex_second
Exposure	Will and Alex decided to throw a party. Will drove Alex to the store. They bought lots of balloons.	Alex_second
Exposure	Will and Alex were bowling. Will loaned some shoes to Alex. Then they picked up two bowling balls.	Alex_second
Exposure	Liz and Alex were in an art class. Liz passed some pigments to Alex. Then they started painting on a canvas.	Alex_second
Critical	Will and Alex were at marching band practice. Will gave some water to Alex. Then they put down the batons.	
Critical	Will and Alex went grocery shopping. Will gave the grocery list to Alex. Then they grabbed a bag of chips.	
Critical	Liz and Alex went fishing. Liz handed a fishing pole to Alex. Then they got out the bait.	
Critical	Liz and Alex went to the farmer’s market. Liz gave a basket to Alex. Then they picked out some tomatoes.	
Critical	Liz and Alex sorted candy after Halloween. Liz passed a chocolate bar to Alex. Then they ate lollipops.	
Critical	Will and Alex went to play soccer. Will handed some cones to Alex. Then they put on their jerseys.	
Critical	Will and Alex went hiking. Will lent a backpack to Alex. Then they packed some snacks.	
Critical	Liz and Alex were studying together. Liz handed some notes to Alex. Then they looked through the textbook.	
Critical	Will and Alex went to a restaurant. Will handed the salt to Alex. Then they ate pasta.	
Critical	Liz and Alex were at a restaurant. Liz handed the menu to Alex. Then they ordered some wine.	
Critical	Will and Alex were deciding what food to make for a party. Will gave an apron to Alex. They made a cake.	
Critical	Liz and Alex went to the store. Liz gave the cart to Alex. Then they bought some soda.	
Critical	Will and Alex were pouring drinks. Will gave a cup to Alex. Then they had some orange juice.	
Critical	Liz and Alex went to a New Year’s Eve party. Liz gave a drink to Alex. Then they popped party poppers.	
Critical	Will and Alex were at the train station. Will gave some money to Alex. Then they bought two tickets.	
Critical	Liz and Alex joined a pottery club. Liz gave some clay to Alex. Then they made vases.	
Critical	Will and Alex were shopping for clothes. Will showed a cool jacket to Alex. Then they decided to buy hats instead.	
Critical	Liz and Alex went to a bakery. Liz lent some money to Alex. Then they bought croissants.	
Critical	Will and Alex were looking at the bookshelves. Will passed an encyclopedia to Alex. Then they rearranged the bookends.	
Critical	Liz and Alex were cleaning up after a dinner party. Liz handed a towel to Alex. Then they dried the plates.	
Filler	Will and Liz wanted to exercise. Will went with Liz to the gym. He ran on the treadmill.	
Filler	Liz and Will played chess. Liz was losing to Will. He captured the king piece.	
Filler	Liz and Will went to a corn maze. Liz rode a tractor with Will. She drank hot chocolate afterwards.	
Filler	Will and Liz went to a farm. Will saw the cows with Liz. Then she fed the goats.	
Filler	Will was fast asleep. Suddenly his alarm went off. He rushed to find his car keys.	
Filler	Liz went to a bar after work. The day had been exhausting. She ordered a cocktail.	
Filler	Will and Liz were at rehearsal. Will helped Liz set up her music stand. Then he got out the sheet music.	
Filler	Will and Liz were bored. Will played a prank on Liz. She sat on a balloon.	
Filler	Will was getting ready for a job interview. He practiced in front of a mirror. Then he put on a tie.	
Filler	Liz was driving home from work. It was foggy and hard to see. She swerved to avoid a deer.	
Filler	Will and Liz went to the airport. Will went through security with Liz. Then he got some coffee.	
Filler	Liz and Will went to the pool. Liz jumped in the water with Will. Then he put on his goggles.	
Filler	Liz and Will were in class. Liz discussed the lecture topics with Will. Then she sharpened a pencil.	
Filler	Will and Liz went to an art museum. Will admired some photography with Liz. She liked the paintings better.	
Filler	Liz was shopping at a jewelry store. None of the gift sets were affordable. So she only bought a necklace.	
Filler	Liz was taking a flight. The pilot warned about some turbulence. She put on some headphones to distract herself.	
Filler	Liz and Will joined an orchestra. Liz practiced with Will. He broke one of the strings on his cello.	
Filler	Liz and Will were doing some chores. Liz spent all day cleaning the apartment with Will. She loved using the new vacuum.	
Filler	Will was knitting a sweater. It was looking a bit too small. He grabbed some more yarn.	
Filler	Will was folding laundry. A spider crawled nearby. He hit it with a shoe.	

## APPENDIX B: DEMOGRAPHIC QUESTIONS IN EXPERIMENT 2

### Pre-Survey Questions

1. What is your age? (drop-down menu provides age options, e.g., 16, 17, 18, 19, 20). The survey automatically ends if the participant puts an age lower than 18.
2. How well do you speak English? (native, good, poor)
3. What age did you start learning English? (baby, before age 6, during elementary school, during high school, during or after college)
4. Do you have normal vision (with or without glasses/contacts)? (yes/no)
5. For auditory experiments only: Is your hearing normal as far as you know? (yes/no)
6. Besides English do you speak any other languages well enough to hold a conversation (yes/no)
  - If yes: What other language do you know best? (write-in)
7. Do you have a language disorder? (yes/no)
  - If yes: Please describe your personal language disorder (write-in)
8. What is the highest level of education you have reached? (less than high school; high school; some college, technical college or a 2-year college; 4-year college; graduate or professional school, other, do not wish to report)
  - If other: What is your highest level of education? (write-in)
9. What gender do you identify with? (woman, man, transgender woman, transgender man, nonbinary, gender-queer, agender, prefer not to say, other, do not wish to report)
  - If other: What is your gender identity? (write-in)
10. What pronouns do you ask other people to use when they talk about you? (she/her; he/him; they/them; other; do not wish to report)
  - If other: What are your personal pronouns? (write-in)
11. What is your ethnicity? (Hispanic or Latino/Latina; Not Hispanic or Latino/Latina; Do not wish to report)
12. What is your race? (Native American / Alaska Native; Asian; Native Hawaiian or Other Pacific Islander; Black or African American; White; More than one race; Unknown; Do not wish to report; Other)
  - If other: What is your race? (write in)

### Post-Survey Questions

13. How many individuals with a non-binary gender identity do you know? (None; 1 or 2; 3 or 4; 5 or more; do not wish to report)
14. How familiar are you with individuals who have a non-binary gender identity?
  - I have never met anyone who identifies as non-binary or has a non-traditional gender identity.
  - I have heard of people in my community (for example at work or in my classes) who identify as non-binary or have a non-traditional gender identity, but I don’t know those individuals well.
  - I have one or two close acquaintances/friends who identify as non-binary or have a non-traditional gender identity.
  - I have three or more close acquaintances/friends who identify as non-binary or have a non-traditional gender identity.
  - Do not wish to report
15. How many people do you know who use *they/them* as personal pronouns, including people who might use multiple pronouns, like *they*/*she* or *they*/*he*?
  - I have never heard of anyone ever using they/them pronouns.
  - I have heard of people on TV or social media using they/them pronouns but I don’t know anyone personally.
  - I have heard of people in my community (for example at work or in my classes) who use they/them pronouns, but I don’t know those individuals well.
  - I have one or two close acquaintances/friends who use they/them pronouns.
  - I have three or more close acquaintances/friends who use they/them pronouns.
16. How often do you use *they*/*them* pronouns in speech or writing to refer to a person whose personal pronouns are *they*/*them*?
  - Never
  - On occasion
  - On average about once or twice a month
  - On average a few times a week
  - On average once a day or more
17. How often do you hear or read *they*/*them* pronouns used to refer to a person whose personal pronouns are *they*/*them*?
  - Never
  - On occasion
  - On average about once or twice a month
  - On average a few times a week
  - On average once a day or more

## APPENDIX C: MODEL ESTIMATES FOR EXPLORATORY ANALYSES

**Table C1.** Inferential statistics for Target Location effects on Responses in Exp. 1.

**Effect**	**Estimate (St. Error)**	***t* Value**	***p***
Intercept	−0.71 (0.23)	−3.08	0.0065
Singular_Prime	1.66 (0.36)	4.62	<.0001
Target_Left	−0.66 (0.31)	−2.11	0.0351
Target_Top	−0.21 (0.33)	−0.63	0.5275
Target_Left * Target_Top	0.12 (0.60)	0.21	0.8354
Singular_Prime * Target_Left	0.14 (0.27)	0.51	0.6115
Singular_Prime * Target_ Top	0.45 (0.27)	1.67	0.0958
_Prime * _Left * _Top	0.41 (0.54)	0.77	0.4436

**Table C2.** Inferential statistics for Order effects on Responses in Exp. 1.

**Effect**	**Estimate (St. Error)**	***t* Value**	***p***
Intercept	−1.11 (0.36)	−3.09	0.0063
Singular_Prime	1.24 (0.42)	2.96	0.0031
Order	0.04 (0.03)	1.57	0.1161
Singular_Prime * Order	0.04 (0.02)	1.82	0.0692

**Table C3.** Inferential statistics for Target Location effects on RTs in Exp. 1.

**Effect**	**Estimate (St. Error)**	***t* Value**	***p***
Intercept	2434.02 (144.87)	16.80	<.0001
Singular_Prime	70.60 (193.31)	0.37	0.7159
Singular_Response	13.02 (94.74)	0.14	0.8917
Target_Left	38.97 (205.64)	0.19	0.8518
Target_Top	53.39 (226.52)	0.24	0.8170
Target_Left * Target_Top	−341.22 (381.23)	−0.90	0.3861
Singular_Prime * Target_Left	−73.57 (131.16)	−0.56	0.5809
Singular_Prime * Target_Top	27.72 (125.40)	0.22	0.8276
Singular_Response * Target_Left	243.29 (176.56)	1.38	0.1829
Singular_Response * Target_Top	70.29 (174.65)	0.40	0.6922

**Table C4.** Inferential statistics for Order effects on RTs in Exp. 1.

**Effect**	**Estimate (St. Error)**	***t* Value**	***p***
Intercept	2736.35 (191.64)	14.28	<.0001
Singular_Prime	130.89 (229.32)	0.57	0.5693
Singular_Response	568.90 (199.82)	2.85	0.0046
Prime * Response	−425.02 (259.73)	−1.64	0.1025
Order	−43.26 (11.86)	−3.65	0.0010
Order * Singular_Prime	7.43 (12.11)	0.61	0.5421
Order * Singular_Response	−35.05 (14.99)	−2.34	0.0195
Order * Prime * Response	7.93 (19.92)	0.40	0.6908

**Table C5.** Inferential statistics for Target Location effects on Responses in Exp. 2.

**Effect**	**Estimate (St. Error)**	***t* Value**	***p***
Intercept	−0.65 (0.20)	−3.19	0.0046
Singular_Prime	1.04 (0.31)	3.32	0.0061
Target_Left	−0.15 (0.30)	−0.48	0.6336
Target_Top	−0.13 (0.30)	−0.44	0.6653
Target_Left * Target_Top	−0.28 (0.59)	−0.46	0.6465
Singular_Prime * Target_Left	0.39 (0.32)	1.23	0.2203
Singular_Prime * Target_Top	−0.58 (0.29)	−1.99	0.047
_Prime * _Left * _Top	−1.10 (0.58)	−1.89	0.0584

**Table C6.** Inferential statistics for Order effects on Responses in Exp. 2.

**Effect**	**Estimate (St. Error)**	***t* Value**	***p***
Intercept	−0.96 (0.32)	−2.97	0.0075
Singular_Prime	0.46 (0.43)	1.07	0.3004
Order	0.01 (0.01)	1.24	0.2142
Singular_Prime * Order	0.02 (0.01)	1.96	0.0506

**Table C7.** Inferential statistics for Target Location effects on RTs in Exp. 2.

**Effect**	**Estimate (St. Error)**	***t* Value**	***p***
Intercept	2205.28 (130.22)	16.93	<.0001
Singular_Prime	404.86 (199.25)	2.03	0.0458
Singular_Response	−83.20 (116.62)	−0.71	0.4812
Target_Left	24.60 (179.11)	0.14	0.8925
Target_Top	−37.66 (178.98)	−0.21	0.8360
Target_Left * Target_Top	139.19 (357.64)	0.39	0.7023
Singular_Prime * Target_Left	−46.04 (126.54)	−0.36	0.7204
Singular_Prime * Target_Top	−204.51 (126.05)	−1.62	0.1228
Singular_Response * Target_Left	183.97 (197.37)	0.93	0.3637
Singular_Response * Target_Top	142.82 (196.47)	0.73	0.4768

**Table C8.** Inferential statistics for Order effects on RTs in Exp. 2.

**Effect**	**Estimate (St. Error)**	***t* Value**	***p***
Intercept	2322.99 (212.16)	10.95	<.0001
Singular_Prime	494.82 (258.36)	1.92	0.0582
Singular_Response	498.14 (224.88)	2.22	0.0272
Prime * Response	−432.53 (301.02)	−1.44	0.1514
Order	−10.67 (4.21)	−2.54	0.0159
Order * Singular_Prime	2.69 (4.66)	0.58	0.5660
Order * Singular_Response	−9.03 (5.29)	−1.71	0.0881
Order * Prime * Response	−1.11 (7.11)	−0.16	0.8764

**Table C9.** Inferential statistics for Familiarity effects on Responses in Exp. 2.

**Effect**	**Estimate (St. Error)**	***t* Value**	***p***
Intercept	−0.59 (0.20)	−3	0.007
Singular_Prime	1.06 (0.33)	3.24	0.0046
Familiarity	−0.04 (0.17)	−0.25	0.8064
Singular_Prime * Familiarity	−0.40 (0.34)	−1.17	0.2423

**Table C10.** Inferential statistics for Familiarity effects on RTs in Exp. 2.

**Effect**	**Estimate (St. Error)**	***t* Value**	***p***
Intercept	2258.26 (126.72)	17.82	<.0001
Singular_Prime	462.36 (202.33)	2.29	0.0253
Singular_Response	−95.36 (118.85)	−0.8	0.4278
Famililary	−73.98 (113.24)	−0.65	0.5156
Singular_Prime * Familiarity	−444.77 (223.99)	−1.99	0.0509
Singular_Response * Familiarity	27.43 (96.63)	0.28	0.7775
Prime * Response * Familiarity	127.49 (194.75)	0.65	0.5155

**Table C11.** Estimates probing the interaction of Prime * Familiarity on RTs in Exp. 2.

**Effect**	**Estimate (St. Error)**	***t* Value**	***p***
Familiarity Effect in Plural Prime Condition	−73.98 (113.24)	−0.65	0.5156
Familiarity Effect in Singular Prime Condition	−518.76 (252.06)	−2.06	0.0432

**Table C12.** Inferential statistics for exploratory analysis for Exposure Effect on responses restricted to critical trials preceded by Alex_Second exposure stories in Experiment 1.

**Effect**	**Estimate (St. Error)**	***t* Value**	***p***
Intercept	−0.52 (0.29)	−1.83	0.10
Singular_Exposure	1.66 (0.37)	4.45	<.0001

**Table C13.** Inferential statistics for exploratory analysis for Exposure Effect on responses restricted to critical trials preceded by Alex_Second exposure stories in Experiment 2.

**Effect**	**Estimate (St. Error)**	***t* Value**	***p***
Intercept	−0.62 (0.23)	−2.7	0.03
Singular_Exposure	0.89 (0.33)	2.71	0.0099

## APPENDIX D: FAMILIARITY QUESTIONNAIRE

**Table D1.** Responses to questions about familiarity with singular *they* and nonbinary gender identities.

	**# respondents**
13. How many individuals with a non-binary gender identity do you know?	
• None	35
• 1 or 2	37
• 3 or 4	3
• 5 or more	4
• do not wish to report	1
14. How familiar are you with individuals who have a non-binary gender identity?	
• I have never met anyone who identifies as non-binary or has a non-traditional gender identity.	10
• I have heard of people in my community (for example at work or in my classes) who identify as non-binary or have a non-traditional gender identity, but I don’t know those individuals well.	36
• I have one or two close acquaintances/friends who identify as non-binary or have a non-traditional gender identity.	29
• I have three or more close acquaintances/friends who identify as non-binary or have a non-traditional gender identity.	4
• Do not wish to report	1
15. How many people do you know who use they/them as personal pronouns, including people who might use multiple pronouns, like they/she or they/he?	
• I have never heard of anyone ever using they/them pronouns.	7
• I have heard of people on TV or social media using they/them pronouns but I don’t know anyone personally.	22
• I have heard of people in my community (for example at work or in my classes) who use they/them pronouns, but I don’t know those individuals well.	23
• I have one or two close acquaintances/friends who use they/them pronouns.	24
• I have three or more close acquaintances/friends who use they/them pronouns.	4
16. How often do you use they/them pronouns in speech or writing to refer to a person whose personal pronouns are they/them?	
• Never	26
• On occasion	26
• On average about once or twice a month	10
• On average a few times a week	12
• On average once a day or more	6
17. How often do you hear or read they/them pronouns used to refer to a person whose personal pronouns are they/them?	
• Never	17
• On occasion	29
• On average about once or twice a month	13
• On average a few times a week	17
• On average once a day or more	4
